# Presence of Selected Methanogens, Fibrolytic Bacteria, and Proteobacteria in the Gastrointestinal Tract of Neonatal Dairy Calves from Birth to 72 Hours

**DOI:** 10.1371/journal.pone.0133048

**Published:** 2015-07-17

**Authors:** Cesar E. Guzman, Lara T. Bereza-Malcolm, Bert De Groef, Ashley E. Franks

**Affiliations:** Department of Physiology, Anatomy and Microbiology, La Trobe University, Melbourne, Victoria, Australia; University of Wisconsin-Madison, UNITED STATES

## Abstract

The microbial communities in the gastrointestinal tract of a young calf are essential for the anatomical and physiological development that permits a transition from milk to solid feed. Selected methanogens, fibrolytic bacteria, and proteobacteria were quantified in the rumen fluid and tissue, abomasum fluid, cecum fluid and tissue, and feces of Holstein bull calves on day 0 (0–20 mins after birth), day 1 (24 ± 1 h after birth), day 2 (48 ± 1 h after birth), and day 3 (72 ± 1 h after birth). Methanogens, fibrolytic bacteria, and *Geobacter* spp. were found to be already present from birth, indicating that microbial colonization of the gastrointestinal tract occurred before or during delivery. The abundance of methanogens and *Geobacter* spp. differed between the days tested and between compartments of the digestive tract and feces, but such difference was not observed for fibrolytic bacteria. Our findings suggests that methanogens might have an alternative hydrogen provider such as *Geobacter* spp. during these early stages of postnatal development. In addition, fibrolytic bacteria were present in the rumen well before the availability of fibrous substrates, suggesting that they might use nutrients other than cellulose and hemicellose.

## Introduction

At the moment of birth, the rumen of a calf has been reported to be sterile and not yet functional [1]. The rumen is then rapidly colonized by microorganisms in the days after birth [2,3]. In their first 3 days of life, the dietary requirements of the calf are fulfilled by the uptake of colostrum only, which is enzymatically digested in the abomasum and the small intestine, and the resulting nutrients are then absorbed in the small intestine. The colostrum provides energy, essential nutrients, and passive immunity to the calf [4,5]. At this stage, the rumen is underdeveloped and virtually no solid feed is consumed during the first 3 days of life [6]. Fibrolytic bacteria are critical for the rapid anatomical and physiological development of the rumen as the new-born calf changes from a liquid diet (colostrum and milk) to solid feed (such as grass and hay) [3,7]. Fibrolytic microbes begin the process of fermentation when the first solid feed is taken up and the products of this fermentation process, such as volatile fatty acids (VFAs; acetic, propionic, and butyric acids), stimulate development of the rumen papillae (rumen tissue) that increase absorption rates of VFAs into the blood. The VFAs supply the metabolic energy required by the calf for essential processes such as growth, thermoregulation, and immunity [6,8].

Three largely unexplored aspects of the symbiosis between bovine ruminants and microbes are the very early colonization of the gastrointestinal tract (GIT) and feces, the development of the microbial communities in the days after birth, and their distribution in the fluid and tissue fractions of GIT components. Using traditional techniques of microbial cultivation and molecular techniques, studies of the rumen fluid in lambs found the first presence of methanogens at 2 days [9,10]. Similar results have been obtained for fibrolytic bacteria [11,12], and more recently, studies using molecular techniques have reported the presence of fibrolytic bacteria and Proteobacteria in the rumen fluid of calves at 1 and 2 days after birth [2,3].

The aim of this study was to determine the abundance of specific microbial species in the GIT (rumen fluid and tissue, abomasum fluid, cecum fluid and tissue, and feces) of calves at 0, 1, 2, and 3 days after birth. We focused on species of microorganisms that have previously been detected in the GIT of 7-day-old calves [13]: methanogens (*Methanomicrobiales mobile*, *Methanococcales voltae*, and *Methanobrevibacter* spp.), proteobacteria (*Geobacter* spp.), and fibrolytic bacteria (*Fibrobacter succinogenes*, *Ruminococcus flavefaciens*, and *Prevotella ruminicola*).

## Materials and Methods

### Animals, diets, and experimental design

The experiment was approved by the Animal Ethics Committee of La Trobe University, Melbourne, Australia (AEC12-32). Twelve Holstein bull calves, all born naturally, were used in total. Samples were collected on day 0 (0–20 mins after birth), day 1 (24 ± 1 h after birth), day 2 (48 ± 1 h after birth), and day 3 (72 ± 1 h after birth). Five days before the delivery date the cow was separated from the herd and placed into a covered area for continuous monitoring. When parturition initiated, the vagina, anus, tail, and legs of the cow were washed with sterile water and then dried with sterile towels. The floor was covered with sterile towels and the calf was received into sterile towels. The calves euthanized on day 0 were separated from their mother immediately after birth and did not consume colostrum prior to euthanization. The calves euthanized on day 1, 2, and 3 were separated from their mother immediately after birth, weighed and fed colostrum. The average body weight of these calves was 40.2 ± 1.3 kg. The calves euthanized on day 1, 2, and 3 had ad-libitum access to water and were fed with colostrum twice daily at 8 am and 4 pm, provided at 15% of the average body weight (6 L/day). The chemical composition of the colostrum calculated on a dry-matter (DM) basis was 210 g DM/kg fresh colostrum, 360 g crude protein/kg DM, 210 g crude fat/kg DM, 61.8 g ash/kg DM, and 256 g lactose/kg DM (DTS Food Laboratories, Melbourne, Australia). The calves were fed colostrum using individual Milkmaid feeders (Polymaster, Melbourne, Australia) fitted with individual Single Peach Teats (Skellerup Industries, Melbourne, Australia). The calves euthanized on days 1, 2, and 3 were housed 3 per 2.5 m × 2.5 m pen in a covered shed with a concrete floor without any sawdust or wood shavings.

### Sample procedures

The calves were euthanized with 20 mL of a single dose of Euthanyl (240 mg/mL; Sigma-Aldrich, Castle Hill, New South Wales, Australia) by intravenous injection into the jugular vein, and all actions minimized suffering. The calves were checked for the absence of breathing, heartbeat and blinking response (corneal reflex) to confirm their death, and then samples were taken within 20 mins. The abdomen was opened and each GIT compartment (rumen, abomasum, cecum, and rectum) was tied with sterile surgical thread at the start and at the end to avoid mixing the contents, and then separated. Each compartment was longitudinally incised along the dorsal line using sterile equipment for each sample. The contents of each compartment were collected and weighed, and then each empty compartment was weighed. Samples consisted of rumen fluid, rumen tissue, abomasum fluid, cecum fluid, cecum tissue, and feces (or meconium for day-0 calves). Triplicate samples were collected from 3 calves per time point resulting in 54 samples per day (3 calves × 6 GIT samples × 3 replicates). Fluid samples from the rumen, cecum, and abomasum were filtered through two layers of sterile cheesecloth and stored in sterile tubes according to Stevenson and Weimer [14]. Tissue samples (2 cm^2^) from the rumen were taken in the dorsal area, and tissue samples from the cecum were taken 5 cm posterior to the ileocecal valve. All tissue samples were rinsed three times with sterilized phosphate-buffered saline (PBS) (pH 7.0) to remove the non-adherent bacteria, according to Li et al. [7]. Pellets of meconium and feces (100 g) were taken by severing the rectum 5 cm from the anus, and then the pellets were rinsed three times with sterile PBS solution according to Yu and Morrison [15]. The samples were transported on dry ice in sterile, airtight plastic tubes within sealed, airtight plastic bags to the laboratory where they were processed immediately.

### DNA extraction

DNA was extracted from 200 mg of each sample of rumen tissue, rumen fluid, abomasum fluid, cecum tissue, cecum fluid, and feces. DNA was extracted using the ZR Fungal/Bacterial DNA MiniPrep Kit and ZR Fecal DNA MiniPrep Kit (Zymo Research, Melbourne, Australia) following the manufacturer’s instructions. The concentration and purity of the extracted DNA samples were assessed spectrophotometrically by measuring the absorbance (A) ratio (A_260_/A_280_) using a Nanodrop 1000 (Thermo Fisher Scientific, Waltham, MA, USA).

### Primers and real-time qPCR

Real-time polymerase chain reaction (qPCR) primers were used to identify the presence of 16S rDNA sequences of *M*. *mobile*, *M*. *votae*, and *Methanobrevibacter* spp. [9]; *Geobacter* spp. [16]; *F*. *succinogenes* [14]; and *R*. *flavefaciens* and *P*. *ruminicola* [17]. The primers were obtained from Geneworks (Adelaide, Australia) (Table 1). To confirm the specificities of the primer pairs, standard PCR reactions were conducted by cloning the 16S rDNA of each species or genus as a template control. After confirming the specific amplification of a DNA fragment of the correct size on agarose gel (Table 1), the PCR products were excised from the gel, purified using the QIAquick Gel Extraction Kit (QIAGEN, Melbourne, Australia), and cloned into the pGEM-T Easy vector (Promega, Melbourne, Australia). Competent *Escherichia coli* JM109 cells were transformed with the ligation products following the manufacturer’s instructions. Plasmids were purified from the transformed *E*. *coli* using a QIAprep Spin Miniprep Kit (QIAGEN) and the plasmid product was sent for sequencing at the Australian Genome Research Facility. The National Center for Biotechnology Information (NCBI) basic local alignment search tool (BLAST) was used to analyse whether the plasmids contained the correct inserts, and to confirm the identity of the microorganisms targeted by the primers (Table 1).

**Table 1:** Sequences of primers used for qPCR detection of methanogens and bacteria. F: forward primer; R: reverse primer.

Target microorganisms	Forward or reverse primer	Primer sequence (5' to 3')	Annealing temperature (°C)	Product size (bp)	Reference
*Methanomicrobiales mobile*	F	TTCYGGTTGATCCYGCCRGA	65	185	[9]
	R	GCGGTGTGTGCAAGGAGC	65		
*Methanoccocales votae*	F	TTCYGGTTGATCCYGCCRGA	65	167	[9]
	R	WASTVGCAACATAGGGCACGG	65		
*Methanobrevibacter* spp.	F	CTCCGCAATGTGAGAAATCG	62	175	[9]
	R	GCGGTGTGTGCAAGGAGC	62		
*Geobacter* spp.	F	AGGAAGCACCGGCTAACTCC	54	320	[16]
	R	TACCCGCRACACCTAGT	54		
*Fibrobacter succinogenes*	F	GCGGGTAGCAAACAGGATTAGA	58	208	[14]
	R	CCCCCGGACACCCAGTAT	58		
*Ruminococcus flavefaciens*	F	CGAACGGAGATAATTTGAGTTTACTTAGG	56	272	[17]
	R	CGGTCTCTGTATGTTATGAGGTATTACC	56		
*Prevotella ruminicola*	F	GCGAAAGTCGGATTAATGCTCTATG	59	215	[17]
	R	CCCATCCTATAGCGGTAAACCTTTG	59		
General bacteria	F	CGGCAACGAGCGCGAACCC	57	130	[19]
	R	CCATTGTAGCACGTGTGTAGCC	57		

Real-time qPCR was performed in triplicate using a Stratagene Mx 3000P qPCR System (Agilent Technologies, Melbourne, Australia). Each reaction mixture (20 μL final volume) contained 10 μL of Brilliant II SYBR Green qPCR Master Mix (Stratagen, Melbourne, Australia), 0.4 μL which contained 10 μM of each primer, and 20 ng of extracted DNA. A no-template sample was included as a negative control to verify that no contaminating nucleic acid was introduced into the master mix or into samples. Positive control samples contained plasmid DNA for each microorganism as a template control.

Real-time qPCR amplification conditions were as follows: an initial denaturation at 95°C for 10 min, followed by 40 cycles of denaturation at 95°C for 30 s, and various annealing temperatures (Table 1) for 30 s, and extension at 72°C for 1 min. A final melting-curve analysis was carried out by continuously monitoring the fluorescence between 55°C and 95°C with 0.5°C increments every 8 s.

Absolute quantification (copy number per μL) was determined using a serial 10-fold dilution from 10^−1^ to 10^−11^ to generate a calibration curve using pure DNA from *M*. *mobile*, *M*. *votae*, *Methanobrevibacter* spp., obtained from the Institute of Agricultural Sciences, Zurich, Switzerland, and *Geobacter* spp. obtained from the University of Massachusetts Amherst, Amherst, USA. Relative quantification of the fibrolytic bacteria (*F*. *succinogenes*, *R*. *flavefaciens*, and *P*. *ruminicola*) was conducted using the delta cycle threshold [ΔC_T_ = C_T_ (target microorganism)–C_T_ (general bacteria)] [18,19].

Amplification efficiencies for each primer pair were investigated by examining the dilution series (from 10^−1^ to 10^−5^) of a pooled DNA template in triplicate and plotting the observed threshold cycle (C_T_) values against the logarithm of total DNA concentration. Values of slopes (ranging from −3.37 to −3.67) and regression coefficients (0.99) were similar to those previously reported for the same primers by Denman and McSweeney (2006), and PCR efficiencies ranged from 95.9% to 98.0%. In this experiment we did not measure feed intake or concentrations of VFAs.

### Statistical analysis

The treatment means were compared one-way ANOVA with post-hoc multiple comparisons using Tukey’s HSD test. All statistical analyses were conducted using SPSS version 22.0 software. Results were considered significant at the *P* < 0.05 level.

## Results

At day 0, which is earlier than in previous studies, we detected methanogens (*M*. *mobile*, *M*. *votae*, and *Methanobrevibacter* spp.), fibrolytic bacteria (*F*. *succinogenes*, *R*. *flavefaciens*, and *P*. *ruminicola*), and *Geobacter* spp. (phylum Proteobacteria) in the rumen fluid, tissues, abomasum fluid, cecum fluid, tissues and feces of calves.

### Rumen fluid and tissue

**Fig 1:**
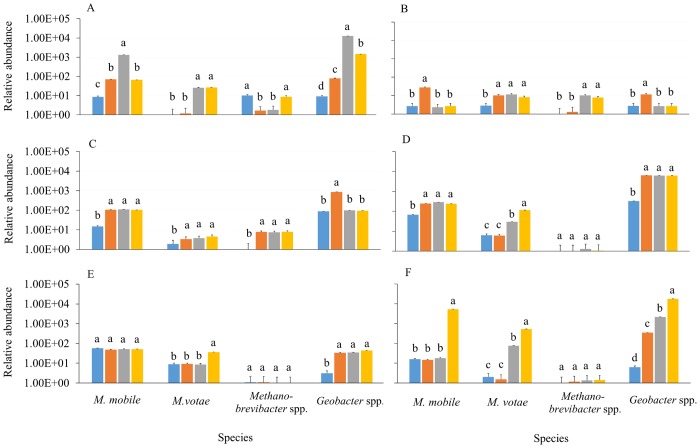
Relative abundance of different species of microorganisms in different parts of the digestive tract of calves during the first four days after birth. The abundance of *Methanomicrobiales mobile*, *Methanococcales votae*, *Methanobrevibacter* spp. and *Geobacter* spp. was determined in (A) rumen fluid, (B) rumen tissue, (C) abomasum, (D) cecum fluid, (E) cecum tissue, and (F) feces of calves of 0 days old (blue bars), 1 day old (orange bars), 2 days old (grey bars), and 3 days old (yellow bars). Abundance, as Log_10_ copy number per μl of 16S ribosomal DNA, is expressed relative to the lowest value among all species. Data are means ± SEM (n = 3). For each species, data without a common letter are significantly different (ANOVA, *P* < 0.05). Note the logarithmic scale of the y-axis.

The abundance of methanogens and *Geobacter* spp. was different between the rumen fluid and rumen tissues. In the rumen fluid, *Geobacter* spp. were more abundant than methanogens on days 2 and 3 (*P* < 0.05) (Fig 1A, S1 Table). The abundance of *M*. *mobile* was higher than that of *M*. *votae* and *Methanobrevibacter* spp. on days 1–3 (*P* < 0.05) (Fig 1A). Across the days, the abundance of each methanogen and *Geobacter* spp. changed whereby *M*. *mobile* was significantly higher in abundance on day 2 than day 0; *M*. *votae* was higher in abundance on day 3 than day 0, and *Methanobrevibacter* spp. were higher in abundance on day 0 than day 1 (*P* < 0.05) (S1 Table). *Geobacter* spp. were more abundant on day 2 than day 0 (*P* < 0.05) (S1 Table). In rumen tissues, the abundance of methanogens was higher or similar to *Geobacter* spp. on days 0–3 (*P* < 0.05) (Fig 1B). The abundance of each methanogen differed across the days. For example, *M*. *mobile* was higher in abundance on day 1 than day 2; *M*. *votae* abundance was higher on day 2 than day 0; and *Methanobrevibacter* spp. were more abundant on day 2 than day 0 (*P* < 0.05). *Geobacter* spp. were more abundant on day 1 than day 2 (*P* < 0.05). *Fibrobacter succinogenes*, *R*. *flavefaciens*, and *P*. *ruminicola* showed no significant difference in abundance between 0, 1, 2, and 3 days in the rumen fluid and in the rumen tissues (*P* < 0.05) (S2 Table).

### Abomasum fluid

In this compartment *M*. *mobile* and *Geobacter* spp. abundances were greater than *M*. *votae* and *Methanobrevibacter* spp. (*P* < 0.05) (Fig 1C). Like for rumen fluid, *M*. *mobile* was higher in abundance on day 2 than day 0; *M*. *votae* was higher on day 3 than day 0 and the abundance of *Geobacter* spp. was higher on day 2 than day 0 (*P* < 0.05; S1 Table). However, in contrast to rumen fluid *Methanobrevibacter* spp. were more abundant on day 3 than day 0 (*P* < 0.05) in abomasum fluid. The fibrolytic bacteria showed no significant difference in abundance between 0, 1, 2, and 3 days in the abomasum fluid (*P* < 0.05) (S2 Table).

### Cecum fluid and tissue

The abundance of the methanogens relative to *Geobacter* spp. differed in the cecum fluid. *Geobacter* spp. were higher in abundance than all methanogens on all days (*P* < 0.05) (Fig 1D). *Methanomicrobiales mobile* and *Geobacter* spp. abundance were higher than *M*. *votae* and *Methanobrevibacter* spp. on days 0–2 (*P* < 0.05) (Fig 1D). *Methanobrevibacter* spp. were lower in numbers than all other measured microorganisms in the cecum fluid and cecum tissues (*P* < 0.05) (Fig 1D). In the cecum fluid, the abundance of *M*. *mobile* was higher on day 2 than day 0; *M*. *votae* was higher on day 3 than day 1 (*P* < 0.05), but there was no significant difference between days for *Methanobrevibacter* spp. (S1 Table). *Geobacter* spp. were significantly more abundant on day 3 than day 0 (*P* < 0.05) (S1 Table). In the cecum tissue, microbial abundances differed to those in the cecum fluid, whereby *Geobacter* spp. were not more abundant than the methanogens on all days (*P* < 0.05) (Fig 1E). The abundance of *M*. *mobile* was not significantly different between the days, *M*. *votae* numbers were higher on day 3 than day 2, and *Methanobrevibacter* spp. were not significantly different between the days. *Geobacter* spp. were significantly more abundant on day 3 than day 2 (*P* < 0.05). The abundance of fibrolytic bacteria did not significantly differ between the days in the cecum fluid and in the cecum tissues (*P* < 0.05) (S2 Table).

### Feces

*Methanomicrobiales mobile* and *Geobacter* spp. were generally more abundant than the other methanogens (*P* < 0.05) (Fig 1F). *Methanobrevibacter* spp. were lower in numbers than all other methanogens (*P* < 0.05) (Fig 1F). The abundances of *M*. *mobile*, *M*. *votae*, and *Geobacter* spp. were higher in the feces on day 3 than on the other days (*P* < 0.05), and the abundance of *Methanobrevibacter* spp. and fibrolytic bacteria in the feces were generally not significantly different between the days (*P* < 0.05; S1 and S2 Tables).

## Discussion

Less than 20 minutes after birth, which is earlier than in previous studies, we detected methanogens (*M*. *mobile*, *M*. *votae*, and *Methanobrevibacter* spp.), fibrolytic bacteria (*F*. *succinogenes*, *R*. *flavefaciens*, and *P*. *ruminicola*), and *Geobacter* spp. (phylum Proteobacteria) in the GIT and feces of calves. This suggests that these organisms start to colonize the GIT as soon as the animal is born, during delivery or even before birth. It was assumed for decades that the human fetus and GIT are sterile at birth, and that during birth the neonatal GIT acquires microorganisms from the maternal vagina and/or from maternal skin and the surrounding environment [20,21]. However, there are now several studies indicating that colonization of the human fetus and GIT by beneficial (non-pathogenic) microbes begins before birth in the placental tissue [22], umbilical cord blood [23], amniotic fluid [24], fetal membranes [25], and meconium [26]. The human fetus swallows some amniotic fluid from the late second trimester of pregnancy [27]. This suggests that microbes present in the amniotic fluid could lead to the microbial colonization of the fetal gut, although the mechanism is not fully understood. The microbes in the amniotic liquid might enter via the placenta, the microbiome of which notably shows similarities to the oral microbiome [22]. The human placental microbiome was found to be composed of Firmicutes, Tenericutes, Proteobacteria, Bacteroidetes, and Fusobacteria, which was similar (at the phylum level) to the oral microbiome but differed from the microbiome of the human skin, vagina, ear, and gut [22]. This suggests that the microorganisms can transfer through the gums in the oral cavity into the bloodstream to colonize the placenta, amniotic liquid and GIT of the fetus before the neonate is born. While the precise mechanisms of microbial colonization of the human fetus are still unclear, similar mechanisms may be responsible for the prenatal microbial colonization of the calf.

There are no previous reports of the presence of the methanogenic species that we examined in the fluid and tissue fractions of the developing rumen in calves prior to 3 days of age. Guzman et al. found the same methanogens in rumen fluid and rumen tissue of 7-day-old calves [13], and other studies have reported the presence of *Methanobrevibacter* spp. and *Methanobacterium* spp [9], and methanogens in general [10], in rumen fluid of young lambs 2 days after birth. Furthermore, the methanogens in the rumen changed in abundance in the first three days of life. It is unlikely that these organisms responded directly to the diet of colostrum because the colostrum and any milk pass directly into the abomasum via the oesophageal groove, which is an anatomical structure resembling a channel [28]. It is more likely that the methanogens in the rumen responded to hydrogen. Methanogens obtain their energy for growth via methanogenesis [29], which requires hydrogen to reduce carbon dioxide, formate, or acetate to methane [30]. Our results raise the question: what is the source of hydrogen for these methanogens between days 0 and 3? In mature ruminants, archaea have been found to associate with protozoa (such as *Entodinium* spp.) because they provide a constant supply of hydrogen and thus significantly contribute to ruminal methanogenesis [31]. However, protozoa have been found to initially establish in the rumen of calves at 21 days and to become fully established at 59 days [32]. Therefore, at 0 to 3 days, this relationship between methanogens and protozoa is unlikely, and other microorganisms such as proteobacteria (*Geobacter* spp.), *R*. *flavefaciens*, or other species might supply the hydrogen for methanogenesis. Proteobacteria (*Geobacter* spp.) are the most abundant, and hence possibly the most important microorganisms during the first 3 days of life in the calf rumen. At the phylum level, a study showed that proteobacteria (which includes *Geobacter* spp.) represent 70% of the bacterial community in the ruminal fluid of calves on day 2 [3] and 45% on days 1 to 3 [2]. *Geobacter* spp. have previously been reported to be present in the developing rumen of 7-day-old calves [13]. In the present study, we found *Geobacter* spp. in the rumen on days 0 to 3, and they were generally in higher abundance than the methanogens on day 2 and 3, suggesting that this relative abundance of *Geobacter* spp. could potentially supply enough hydrogen to the methanogens. We propose that *Geobacter* spp. could form a syntrophic partnership with methanogens by direct electron transfer and provision of hydrogen for the reduction of organic compounds in the rumen of calves in this early stage of life. *Geobacter* spp. has been shown to use the recently identified mechanism of direct extracellular electron transfer to supply electrons directly to archaea [33,34]. Xu *et al*. found that *Methanobacterium* spp. and *Methanosarcina* spp. have the capacity for directly accepting electrons and hydrogen from *Geobacter* spp. [35] Alternatively, *R*. *flavefaciens* might also supply hydrogen and electrons to the methanogens [36].

The relative abundance of methanogens species in the rumen differs between 0- to 3-day-old calves and mature animals. In mature cows, *Methanobrevibacter* spp. represented 62% of the rumen archaea, and they were among the most important and dominant archaea in the rumen fluid [30,37]. We detected *Methanobrevibacter* spp. in the rumen of neonatal calves, but at lower abundance than *M*. *mobile* and *M*. *votae*, indicating that *Methanobrevibacter* spp. is not the most important methanogen, at least in terms of abundance, in the first 3 days after birth. The reason for this difference is not clear.

In this study, the abundance of fibrolytic bacteria did not differ between the days. Fibrolytic bacteria digest solid fiber (i.e., cellulose, hemicellulose, and xylan) in the mature ruminant [38], but these nutrients were not present in the calves’ diet at this age. Therefore, colonization of the rumen by these fibrolytic bacteria began before solid fiber was present in the rumen. An interesting question is: what nutrients did the fibrolytic microorganisms use to maintain their population? Our recent study suggested that fibrolytic bacteria in the rumen of 7-day-old calves could obtain nutrients from milk [13]. In addition, other species of microorganisms might provide an alternative supply of nutrients for the fibrolytic bacteria.

In the abomasum fluid, the abundance of methanogens differed between the days, whereas the abundance of fibrolytic bacteria did not change. The methanogens were in lowest abundance compared with the other GIT compartments, which is not surprising given the pH in the abomasum of calves is around 2.0–3.2 [39]. Currently, there are no studies about microorganisms in the abomasum of calves in the first 3 days after birth, which limits a substantial discussion about this topic. A study in mature horses found Proteobacteria, Firmicutes, Actinobacteria, and Bacteroidetes in the stomach [40], indicating that microorganisms capable of surviving in the very acidic environment of the abomasum.

Methanogens, fibrolytic bacteria, and *Geobacter* spp. have been found in the cecum of 7-day-old calves [13], and different concentrations of Bacteroidetes, Firmicutes, and Proteobacteria have been found between cecum ingesta and cecum tissue of 21-day-old calves [41]. Here we report the presence of methanogens, fibrolytic bacteria, and *Geobacter* spp. in the cecum of neonatal calves, with changes in the number of methanogens and *Geobacter* spp. but not of fibrolytic bacteria during the first 3 days of life. Microorganisms in the cecum of mature cattle digest the fiber that was not digested in the rumen and produce fermentation products, mainly VFAs, and vitamins in addition to waste products such as methane [42]. However, 0- to 3-day-old calves do not ingest solid feed; hence they have no fiber in their cecum. This once again suggests that fibrolytic bacteria (and methanogens by extension) may utilize another energy source.

Lastly, methanogens, *Geobacter* spp., and fibrolytic bacteria were already present in the feces on day 0. At this time the feces are known as meconium, which is the earliest stool of a mammalian neonate, different to the subsequent feces. The meconium is composed of materials ingested during gestation such as intestinal epithelial cells, lanugo, mucus, amniotic fluid, bile, and water [26]. Meconium sampled 5 cm anterior to the anus within the rectum might be isolated from contact with microorganisms during the birthing process when the fetus is exposed to rich sources of microbes, such as vaginal secretions from the mother and skin contact. The unborn calf might have ingested microbes during labor, but it is unknown whether these microbes could accumulate in the meconium within 20 mins. The second major microbial exposure to the neonate occurs with the consumption of colostrum. However, the calves were separated from the mother immediately after birth and did not have contact with the mother and did not drink colostrum. We therefore speculate that the presence of methanogens, *Geobacter* spp., and fibrolytic bacteria in the meconium again suggests that the GIT was colonized before the calves were born. Studies in human indicate that the microorganisms in the meconium differ from those observed in early feces. Firmicutes was the main phylum detected in meconium, whereas Proteobacteria (*Geobacter* spp.) was predominant in early feces [26,43]. The presence of these microorganisms in the meconium may result from the human fetus swallowing amniotic liquid during the last three months of pregnancy [27] and a similar process may explain the presence of microbes in the meconium of the calf. After birth, on day 2 and on day 3, the methanogens *M*. *mobile* and *M*. *votae*, and *Geobacter* spp. in the feces showed a 10-fold increase in abundance, suggesting that calf exposure to colostrum and the environment did contribute to microbial growth.

### Conclusions

This study shows that less than 20 minutes after birth, methanogens, *Geobacter spp*., and fibrolytic bacteria are present in the GIT of calves. Moreover, the composition of the microbial community throughout the GIT of calves up to 3 days of age varied in abundance between compartments (rumen, cecum, and abomasum) and feces. Methanogens were detected prior to the colonization by protozoa, suggesting that other microorganisms such as *Geobacter* spp. might play a role in supplying hydrogen and electron transfer. Fibrolytic bacteria present in the rumen and cecum before the calves were capable of consuming and digesting solid fiber, indicates that fibrolytic bacteria might use nutrients other than cellulose, hemicellose, and xylan, such as the nutrients obtained from colostrum or provided by other species of microorganisms. If we understand the microbial colonization of the entire GIT from the time before birth, then we might be equipped to assist its anatomical and physiological development during the transition from milk to solid feed.

## Supporting Information

S1 Table Log_10_ copy number per μL of 16S ribosomal DNA of the methanogens *M*. *mobile*, *M*. *votae*, and *Methanobrevibacter* spp., and *Geobacter* spp. as determined by real-time quantitative PCR in ruminal fluid, ruminal tissue, abomasum, cecum fluid, cecum tissue, and feces of calves at 0, 1, 2, and 3 days of age (means ± se; n = 3). Means within a row followed by a different superscript letter indicate a significant difference (ANOVA, *P* < 0.05).
(DOCX)

S2 Table Threshold cycle values (ΔC_T_) from real-time quantitative PCR allowing semi-quantification of the fibrolytic bacteria *F*. *succinogenes*, *R*. *flavefaciens*, and *P*. *ruminicola* in ruminal fluid, ruminal tissue, abomasum, cecum fluid, cecum tissue, and feces at 0, 1, 2, and 3 days of age (means ± se; n = 3). A lower ΔC_T_ value indicates a higher concentration of the microorganism. There were no significant differences between any of the means (ANOVA, *P* > 0.05).
(DOCX)
