# Supplementary material for: Presence of Selected Methanogens, Fibrolytic Bacteria, and Proteobacteria in the Gastrointestinal Tract of Neonatal Dairy Calves from Birth to 72 Hours
Source: PLoS One. 2015 Jul 17;10(7):e0133048. doi: 10.1371/journal.pone.0133048 (PMC4505879; doi:10.1371/journal.pone.0133048)
Supplement: S1 Table — Means within a row followed by a different superscript letter indicate a significant difference (ANOVA, P < 0.05). (DOCX) [file pone.0133048.s001.docx]

# Supporting Information

**S1 Table. Log_10_ copy number per μL of 16S ribosomal DNA of the methanogens *M. mobile*, *M. votae*, and *Methanobrevibacter* spp., and *Geobacter* spp*.* as determined by real-time quantitative PCR in ruminal fluid, ruminal tissue, abomasum, cecum fluid, cecum tissue, and feces of calves at 0, 1, 2, and 3 days of age (means ± se; n=3).** Means within a row followed by a different superscript letter indicate a significant difference (ANOVA, *P* < 0.05).

| Species | 0 days | 1 day | 2 days | 3 days |
| --- | --- | --- | --- | --- |
| *Methanomicrobiales mobile* | | | | |
| Rumen fluid | (2.02^c^ ± 0.60) x 10^3^ | (1.65^b^ ± 0.66) x 10^4^ | (3.11^a^ ± 0.65) x 10^5^ | (1.54^b^ ± 0.68) x 10^4^ |
| Rumen tissue | (3.83^b^ ± 0.46) x 10^3^ | (3.97^a^ ± 0.46) x 10^4^ | (3.28^b^ ± 0.49) x 10^3^ | (3.81^b^ ± 0.46) x 10^3^ |
| Abomasum | (6.94^b^ ± 0.60) x 10^2^ | (4.87^a^ ± 0.62) x 10^3^ | (5.12^a^ ± 0.61) x 10^3^ | (4.79^a^ ± 0.61) x 10^3^ |
| Cecum fluid | (8.82^b^ ± 0.57) x 10^4^ | (3.21^a^ ± 0.50) x 10^5^ | (3.74^a^ ± 0.55) x 10^5^ | (3.14^a^ ± 0.55) x 10^5^ |
| Cecum tissue | (2.51^a^ ± 0.40) x 10^5^ | (2.15^a^ ± 0.44) x 10^5^ | (2.31^a^ ± 0.41) x 10^5^ | (2.22^a^ ± 0.47) x 10^5^ |
| Feces | (1.86^b^ ± 0.61) x 10^4^ | (1.68^b^ ± 0.62) x 10^4^ | (2.07^b^ ± 0.66) x 10^4^ | (6.33^a^ ± 0.60) x 10^6^ |
| *Methanococcales votae* | | | | |
| Rumen fluid | (2.35^b^ ± 0.32) x 10^2^ | (2.79^b^ ±0.49) x 10^2^ | (5.98^a^ ± 0.27) x 10^3^ | (6.22^a^ ± 0.51) x 10^3^ |
| Rumen tissue | (2.98^b^ ± 0.29) x 10^3^ | (1.48^a^ ± 0.34) x 10^4^ | (1.67^a^ ± 0.16) x 10^4^ | (1.18^a^ ± 0.32) x 10^4^ |
| Abomasum | (8.86^b^ ± 0.45) x 10^1^ | (1.54^a^ ± 0.53) x 10^2^ | (1.75^a^ ± 0.38) x 10^2^ | (2.08^a^ ± 0.54) x 10^2^ |
| Cecum fluid | (8.25^c^ ± 0.29) x 10^3^ | (7.94^c^ ± 0.33) x 10^3^ | (3.88^b^ ± 0.21) x 10^4^ | (1.49^a^ ± 0.42) x 10^5^ |
| Cecum tissue | (3.98^b^ ± 0.54) x 10^4^ | (4.05^b^ ± 0.46) x 10^4^ | (3.78^b^ ± 0.69) x 10^4^ | (1.58^a^ ± 0.22) x 10^5^ |
| Feces | (2.38^c^ ± 0.21) x 10^3^ | (1.81^c^ ± 0.28) x 10^3^ | (8.99^b^ ± 0.36) x 10^4^ | (6.16^a^ ± 0.17) x 10^5^ |
| *Methanobrevibacter* spp. | | | | |
| Rumen fluid | (2.37^a^ ± 0.23) x 10^3^ | (3.90^b^ ± 0.24) x 10^2^ | (4.32^b^ ± 0.52) x 10^2^ | (2.07^a^ ± 0.64) x 10^3^ |
| Rumen tissue | (1.43^b^ ± 0.19) x 10^3^ | (1.84^b^ ± 0.16) x 10^3^ | (1.45^a^ ± 0.23) x 10^4^ | (1.12^a^ ± 0.38) x 10^4^ |
| Abomasum | (4.62^b^ ± 0.22) x 10^1^ | (3.60^a^ ± 0.27) x 10^2^ | (3.44^a^ ± 0.41) x 10^2^ | (3.67^a^ ± 0.54) x 10^2^ |
| Cecum fluid | (1.35^a^ ± 0.20) x 10^3^ | (1.31^a^ ± 0.17) x 10^3^ | (1.69^a^ ± 0.27) x 10^3^ | (1.39^a^ ± 0.32) x 10^3^ |
| Cecum tissue | (4.76^a^ ± 0.24) x 10^3^ | (4.83^a^ ± 0.39) x 10^3^ | (4.39^a^ ± 0.37) x 10^3^ | (4.41^a^ ± 0.42) x 10^3^ |
| Feces | (1.17^a^ ± 0.21) x 10^3^ | (1.37^a^ ± 0.13) x 10^3^ | (1.58^a^ ± 0.72) x 10^3^ | (1.66^a^ ± 0.38) x 10^3^ |
| *Geobacter* spp. | | | | |
| Rumen fluid | (2.18^c^ ± 0.21) x 10^3^ | (1.88^b^ ± 0.38) x 10^4^ | (2.95^a^ ± 0.34) x 10^6^ | (3.42^b^ ± 0.58) x 10^5^ |
| Rumen tissue | (3.92^b^ ± 0.12) x 10^3^ | (1.67^a^ ± 0.26) x 10^4^ | (3.82^b^ ± 0.19) x 10^3^ | (3.83^b^ ± 0.54) x 10^3^ |
| Abomasum | (4.08^b^ ± 0.17) x 10^3^ | (3.98^a^ ± 0.25) x 10^4^ | (4.56^b^ ± 0.64) x 10^3^ | (4.35^b^ ± 0.49) x 10^3^ |
| Cecum fluid | (4.34^b^ ± 0.14) x 10^5^ | (8.31^a^ ± 0.11) x 10^6^ | (8.15^a^ ± 0.49) x 10^6^ | (8.07^a^ ± 0.31) x 10^6^ |
| Cecum tissue | (1.37^b^ ± 0.23) x 10^4^ | (1.49^a^ ± 0.45) x 10^5^ | (1.54^a^ ± 0.31) x 10^5^ | (1.95^a^ ± 0.43) x 10^5^ |
| Feces | (7.34^d^ ± 0.19) x 10^3^ | (4.08^c^ ± 0.37) x 10^5^ | (2.53^b^ ± 0.24) x 10^6^ | (2.04^a^ ± 0.36) x 10^7^ |
