# Supplementary material for: Presence of Selected Methanogens, Fibrolytic Bacteria, and Proteobacteria in the Gastrointestinal Tract of Neonatal Dairy Calves from Birth to 72 Hours
Source: PLoS One. 2015 Jul 17;10(7):e0133048. doi: 10.1371/journal.pone.0133048 (PMC4505879; doi:10.1371/journal.pone.0133048)
Supplement: S2 Table — A lower ΔCT value indicates a higher concentration of the microorganism. There were no significant differences between any of the means (ANOVA, P > 0.05). (DOCX) [file pone.0133048.s002.docx]

# Supporting Information

**S2 Table. Threshold cycle values (ΔC_T_) from real-time quantitative PCR allowing semi-quantification of the fibrolytic bacteria *F. succinogenes*, *R. flavefaciens*, and *P. ruminicola* in ruminal fluid, ruminal tissue, abomasum, cecum fluid, cecum tissue, and feces at 0, 1, 2, and 3 days of age (means ± se; n=3).** A lower ΔC_T_ value indicates a higher concentration of the microorganism. There were no significant differences between any of the means (ANOVA, *P* > 0.05).

| Species | 0 days | 1 day | 2 days | 3 days |
| --- | --- | --- | --- | --- |
| *Fibrobacter succinogenes* | | | | |
| Rumen fluid | 18.14 ± 1.22 | 17.84 ± 1.38 | 17.94 ± 1.25 | 17.94 ± 1.26 |
| Rumen tissue | 17.92 ± 1.26 | 17.81 ± 1.32 | 17.74 ± 1.40 | 17.74 ± 1.27 |
| Abomasum | 17.88 ± 1.27 | 17.55 ± 1.35 | 17.81 ± 1.10 | 17.16 ± 1.12 |
| Cecum fluid | 17.83 ± 1.23 | 17.62 ± 1.43 | 17.73 ± 1.37 | 17.13 ± 1.12 |
| Cecum tissue | 17.78 ± 1.14 | 17.75 ± 1.36 | 17.75 ± 1.61 | 17.75 ± 1.38 |
| Feces | 17.96 ± 1.05 | 18.15 ± 1.22 | 18.16 ± 1.40 | 18.14 ± 1.28 |
| *Ruminococcus flavefaciens* | | | | |
| Rumen fluid | 19.73 ± 1.32 | 19.91 ± 1.21 | 20.26 ± 1.35 | 20.85 ± 1.52 |
| Rumen tissue | 19.57 ± 1.32 | 19.49 ± 1.69 | 19.82 ± 1.22 | 19.12 ± 1.51 |
| Abomasum | 19.51 ± 1.30 | 19.47 ± 1.23 | 19.59 ± 1.24 | 19.16 ± 1.31 |
| Cecum fluid | 19.58 ± 1.15 | 19.18 ± 1.50 | 17.73 ± 1.37 | 18.84 ± 1.19 |
| Cecum tissue | 19.51 ± 1.21 | 19.38 ± 1.57 | 19.65 ± 1.53 | 19.72 ± 1.07 |
| Feces | 19.52 ± 1.32 | 19.39 ± 1.61 | 21.00 ± 1.53 | 20.65 ± 1.37 |
| *Prevotella ruminicola* | | | | |
| Rumen fluid | 20.59 ± 1.41 | 20.87 ± 1.57 | 20.51 ± 1.42 | 20.51 ± 1.65 |
| Rumen tissue | 20.57 ± 1.62 | 20.56 ± 1.42 | 20.80 ± 1.10 | 20.34 ± 1.36 |
| Abomasum | 20.54 ± 1.28 | 20.41 ± 1.48 | 17.81 ± 1.47 | 20.52 ± 1.57 |
| Cecum fluid | 20.05 ± 1.21 | 20.46 ± 1.61 | 19.16 ± 1.51 | 19.76 ± 1.26 |
| Cecum tissue | 20.01 ± 1.34 | 20.82 ± 1.48 | 20.67 ± 1.36 | 20.12 ± 1.25 |
| Feces | 21.24 ± 1.38 | 21.67 ± 1.32 | 20.64 ± 1.25 | 20.88 ± 1.23 |
